# Revisiting targeted therapy and immunotherapy for advanced cholangiocarcinoma

**DOI:** 10.3389/fimmu.2023.1142690

**Published:** 2023-03-01

**Authors:** Jiajia Du, Xing Lv, Zunyi Zhang, Zhiyong Huang, Erlei Zhang

**Affiliations:** Hepatic Surgery Center, Tongji Hospital, Tongji Medical College, Huazhong University of Science and Technology, Wuhan, Hubei, China

**Keywords:** cholangiocarcinoma, targeted therapy, immunotherapy, systemic therapy, PD-1

## Abstract

Cholangiocarcinoma (CCA) is a rare and aggressive type of malignant tumor. In the past few years, there has been an increase in the incidence of CCA. Surgery is the only effective treatment but is only suitable for a small percentage of patients. Comprehensive treatment is the normal therapy for terminal CCA patients, depending basically on gemcitabine and cisplatin combination chemotherapy. In the past decade, the emergence of next-generation sequencing technology can be used for the identification of important molecular features of CCA, and several studies have demonstrated that different CCA subtypes have unique genetic aberrations. Targeting fibroblast growth factor receptor (FGFR), isocitrate dehydrogenase (IDH) and epidermal growth factor receptor 2 (EGFR2) are emerging targeted therapies. In addition, researches have indicated that immunotherapy has a key function in CCA. There is ongoing research on programmed cell death protein 1 inhibitors (PD-1), chimeric antigen receptor T cells (CAR-T) and tumor-infiltrating leukocyte (TILs). Researches have shown that targeted therapy, immunotherapy, and conventional chemotherapy in CCA had certain mechanistic links, and the combination of those can greatly improve the prognosis of advanced CCA patients. This study aimed to review the research progress of targeted therapy and immunotherapy for CCA.

## 1 Introduction

Cholangiocarcinoma (CCA) is the second most common primary liver malignancy, accounting for 3% of digestive tract tumors. The CCA incidence rate ranges from 0.35 to 2 per 100,000 people every year in western countries. However, this incidence rate can be as high as 40 times in China, thereby leading to a major public health concern. CCA is divided into extrahepatic cholangiocarcinoma (eCCA) and intrahepatic cholangiocarcinoma (iCCA), with the former further divided into distal cholangiocarcinoma (dCCA) and perihilar cholangiocarcinoma (pCCA) (1). Although these subgroups differ significantly in prognosis, etiology, biology, and epidemiology (2), surgical treatment is found to be the optimal treatment for localized CCA. iCCA treatment can be performed with hepatectomy based on anatomical conditions, whereas eCCA may be treated with hepatopancreaticoduodenectomy. However, the surgical recurrence rate of CCA can be up to more than 50% despite receiving postoperative adjuvant chemotherapy (3).

Due to the lack of early clinical symptoms, about 75% of CCA patients are identified as metastatic or locally advanced disease at initial diagnosis. Based on the ABC-02 clinical study, cisplatin/gemcitabine (CisGem) is considered the first-line treatment for patients with advanced CCA. According to the ABC-06 study, the standard second-line treatment after CisGem is a fluorouracil plus oxaliplatin (FOLFOX) regimen, however, the objective response rate (ORR) was not statistically significant between the FOLFOX group and the control group (4). Besides, adding nab-paclitaxel (Abraxane) to CisGem did not result in a statistically significant improvement in OS in patients with newly diagnosed advanced CCA in the Phase 3 SWOG 1815 trial (NCT03768414). It is believed that the overall effect of systemic chemotherapy in CCA remains unsatisfactory.

With the enhanced understanding of the molecular pathways in CCA, targeted therapy has currently become one of the most innovative therapeutic approaches, and is primarily used to attack particular genes or proteins playing a crucial part in the carcinogenesis and progression of CCA. In a previous study (5), next-generation sequencing (NGS) was used for mapping CCA, 182 cancer-associated genes and 37 introns were identified from 14 cancer-rearranged genes, which demonstrated that biliary tract tumors share the same chromatin remodeling (ARID1A) and genomic aberrations (CDKN2B). The TME of CCA contains tumor-associated fibroblasts and inhibitory immune components, leading to T cell-mediated rejection, inhibition of anti-tumor immune response, and promotion of tumorigenesis, as well as possibly influencing the mechanism of chemotherapy (6). At present, immune checkpoint inhibitors (ICIs), chimeric antigen receptor T cells (CAR-T), tumor vaccines, and tumor-infiltrating leukocytes (TILs) are the major immunotherapy methods (7–9).

**Figure 1:**
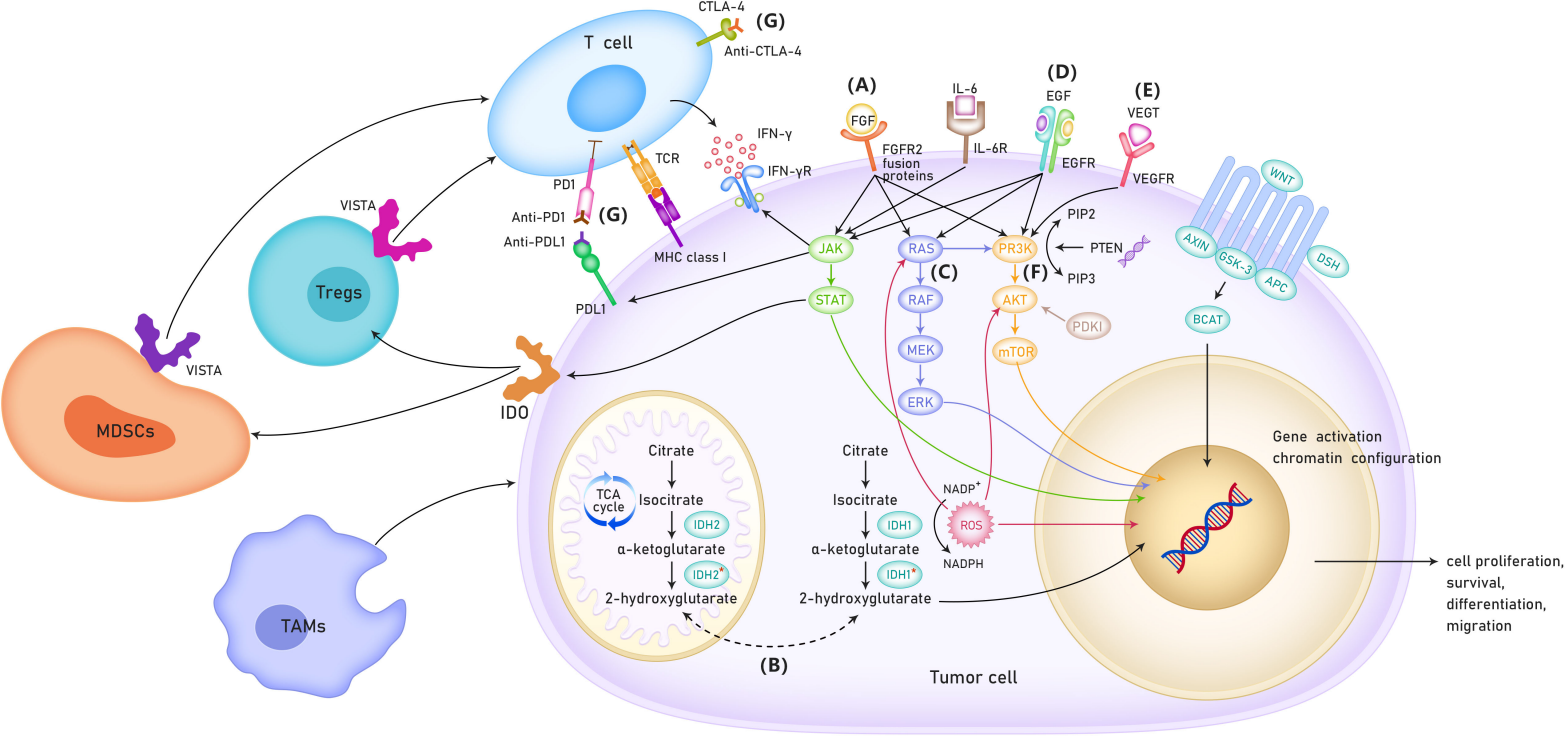
The possible mechanisms of targeted therapy and immunotherapy for cholangiocarcinoma. **(A)** Role of FGFR in the treatment of cholangio- carcinoma. **(B)** Role of IDH 1/2 mutation in the treatment of patients with cholangiocarcinoma. **(C)** RAS/RAF/MEK/ERK signaling pathway. **(D)** ErbB amplifi- cation. **(E)** VEGF inhibitors. **(F)** PI3K/AKT/mTOR signaling pathway. **(G)** Immune checkpoint inhibitors. TCA cycle, TriCarboxylic Acid cycle; VISTA, V-type immunoglobulin domain-containing suppressor of T cell activation; MDSCs, Myeloid-derived suppressor cells; TAMs,tumor-associated macro- phages; IDO, Indoleamine-2,3-Dioxygenase.

It was hypothesized that targeted therapy, immunotherapy, and conventional chemotherapy had synergistic effects in CCA. A combination of targeted therapy, immunotherapy, and conventional chemotherapy may significantly improve the prognosis of CCA. For example, MEK inhibitor monotherapy had limited efficacy in CCA; however, it can enhance the efficacy of PD-L1 inhibitors (10). In addition, the combination of VEGF inhibitors and ICIs have shown enhanced immune activation, increased tumor destruction, and improved efficacy in preclinical models (11). The present systemic review provided an overview of the studies based on the targeted therapy and immunotherapy for advanced CCA. Furthermore, this study also summarized the current status and potential mechanistic links between targeted therapy and immunotherapy (**Figure 1**).

## 2 Targeted therapy

Molecular targets such as FGFR2 fusion, IDH1/2 mutation and HER2 amplification are proposed to be utilized for targeted therapies in CCA, but a majority of them are generally under clinical investigation (**Table 1**).

**Table 1:** Selected clinical trials of targeted therapy in advanced cholangiocarcinoma.

Mutation/Pathway	Agent	Trial	Setting	Patients	Treatment Arm(s)	Primary Endpoint	Grade 3/4 Adverse Events
FGFR	Pemigatinib (FGFR1/2/3)	FIGHT-202 (12)	2nd-line	locally advanced/metastatic CCA with and without FGFR2 fusions/rearrangements	Pemigatinib 13.5mg qd	ORR (35.5%)	hypophosphataemia [12%], arthralgia [6%], stomatitis [5%], hyponatremia [5%], abdominal pain [5%], fatigue [5%]
		FIGHT-302 (13)	2^nd^-line	locally advanced/metastatic CCA with FGFR2 fusions/rearrangements	Pemigatinib (13.5mg qd) vs Gem(1000mg/m^2^)Cis(25mg/m^2^)	PFS	
	Infigratinib (FGFR1/2/3)	Javle M et al., 2018 (14)	2^nd^-line	advanced iCCA with FGFR2 fusions	Infigratinib 125mg qd	cORR (26.9%)	hypophosphatemia [14.1%], hyperphosphatemia [12.7%], hyponatremia [11.3%]
		Javle M et al., 2018 (15)	2^nd^-line	advanced/metastatic CCA with FGFR2 fusions or other FGFR alterations	Infigratinib 125mg qd	ORR (14.8%)	hyperphosphatemia [16.4%], stomatitis [6.6%], palmar-plantar erythrodysesthesia [4.9%]
		Javle M et al., 2021 (3)	2^nd^-line	advanced CCA with FGFR2 fusions/rearrangements	Infigratinib 125mg qd	ORR (23.1%) DOR (5.0 m)	stomatitis [14.8%], hyponatremia [13.0%], hypophosphatemia [13.0%]
		Javle M et al., 2021 (16)	2^nd^-line	advanced or metastatic CCA with FGFR2 fusions or rearrangements	Infigratinib 125mg qd	ORR (23.1%)	hyperphosphataemia (n = 83), stomatitis (n = 59), fatigue (n = 43), alopecia (n = 41), dry eyes (n = 37)
		PROOF 301 (17)	1st-line	advanced CCA with FGFR2 gene fusions/translocations	Infigratinib 125mg qd vs Gem(1000 mg/m^2^)Cis(25 mg/m^2^)	PFS	
	Derazantinib (FGFR1/2/3)	Mazzaferro V et al., 2017 (18)	2nd-line	advanced iCCA with FGFR2 genetic aberrations	Derazantinib 300/400mg qd	ORR	asthenia [6%], abnormal LFTs [6%]
		Mazzaferro V et al., 2019 (19)	2nd-line	advanced or inoperable iCCA with FGFR2 gene fusion-positive	Derazantinib 300mg qd	ORR (20.7%) DCR (82.8%) PFS (5.7 m)	hyperphosphatemia [10.3%], eye toxicity [6.9%], upper gastrointestinal hemorrhage [3.5%]
		Busset M D D et al., 2019 (20)		iCCA expressing FGFR2-fusion, FGFR2 mutations/amplifications, no FGFR gene aberration	Derazantinib 300 mg qd	ORR DCR PFS	
		FIDES-01 (21)	2nd-line	iCCA with FGFR2 mutations or amplifications	Derazantinib 300 mg qd	proportion of pts alive PFS3 (76.3%)	
	Erdafitinib (FGFR1/2/3/4)	Soria J C et al., 2017 (22)	2nd-line	advanced CCA with FGFR gene alterations, including activating mutations and translocations or other FGFR-activating aberrations	Erdafitinib 9 mg qd/10 mg intermittently (7 days on/7 days off)	ORR (27.3%)	stomatitis [18%]
		Bahleda R et al., 2019 (23)	2nd-line	advanced or refractory CCA with activating FGFR genomic alterations	Erdafitinib 9 mg qd/10 mg intermittently (7 days on/7 days off)	ORR (27.3%)	hyperphosphatemia [0.5%]
		Feng Y H et al., 2022 (4)	2nd-line	advanced CCA with FGFR alterations	Erdafitinib 8/9 mg qd	ORR (40.9%)	stomatitis [13.6%], ALT increased [13.6%]
	Futibatinib (FGFR1/2/3/4)	FOENIX-101 (24)	2nd-line	locally advanced or metastatic iCCA with FGFR2 gene rearrangements	Futibatinib 200 mg qd	ORR	
		FOENIX-CCA3 (25)	1st-line	advanced iCCA harboring FGFR2 gene rearrangements	Futibatinib 200 mg qd vs Gem(1000 mg/m^2^)Cis(25 mg/m^2^)	PFS	
		FOENIX-CCA2 (26)	2nd-line	locally advanced/metastatic unresectable iCCA harboring FGFR2 gene fusions or other rearrangements	Futibatinib 20 mg qd	ORR (34.3%)	hyperphosphatemia [25.4%]
		Goyal L et al., 2023 (27)	2nd-line	unresectable or metastatic iCCA with FGFR2 fusion-positive or FGFR2 rearrangement-positive	Futibatinib 20 mg qd	ORR (42%)	hyperphosphatemia [30%], increased aspartate aminotransferase [7%], stomatitis [6%], fatigue [6%]
	debio 1347	Cleary J M et al., 2018 (28)	2nd-line	advanced iCCA/GBC patients with FGFR1/2/3 activating amplifications/mutations/translocations	debio1347 60–150 mg qd	ORR DCR (62.5%)	hyperphosphatemia [4/8]
IDH1/2	Ivosidenib	ClarIDHy (29)	2nd-line	advanced CCA with IDH1-mutant	Ivosidenib 500 mg qd	PFS (2.7 m)	Ascites [7%]
RAS-RAF-MEK-ERK	Adagrasib	KRYSTAL-1 (30)	2nd-line	patients with unresectable or metastatic gastrointestinal (GI) tumors harboring a KRASG12C mutation	Adagrasib 600 mg BID	PR (41%) DCR (100%)	
	Dabrafenib、Trametinib	ROAR II (31)	2nd-line	advanced or metastatic CCA with BRAF V600E–mutant	D (150 mg BID) + T (2 mg qd)	ORR(41%)	increased γ-glutamyltransferase [9%], decreased white blood cell count [9%]
	Selumetinib	Bekaii Saab T et al., 2011 (32)		advanced/metastatic CCA	Selumetinib 100 mg bid	ORR (12%)	fatigue [4%]
	Selumetinib	ABC-04 (33)	2nd-line	advanced/metastatic CCA	Selumetinib 75 mg bid + Gem(1000 mg/m^2^)Cis(25 mg/m^2^)	ORR PFS (6.4 m)	
	Selumetinib	Doherty M et al., 2018 (34)	1st-line	advanced CCA	Selumetinib 50 mg qd + Gem (1000 mg/m^2^)Cis(25 mg/m^2^) (qd) vs Selumetinib 50 mg qd + Gem(1000 mg/m^2^)Cis(25 mg/m^2^) (q21d (1–5, 8–19)) vs Gem(1000 mg/m^2^)Cis(25 mg/m^2^)	%change in RECIST tumor size (p = 0.8)	
	Ulixertinib	Sullivan R J et al., 2018 (35)		advanced CCA with MAPK (BRAF, MEK) mutant	Ulixertinib 10–900 mg/600 mg qd	ORR	
NTRK	Entrectinib	Doebele RC et al.,2020 (36)		advanced or metastatic CCA with NTRK fusion-positive	Entrectinib 600 mg qd	ORR (57%) mDOR	increased weight [10%], anemia [12%], nervous system disorders [4%]
	Larotrectinib	Hong DS et al., 2020 (37)	2nd-line	locally advanced or metastatic CCA with TRK fusion-positive	Larotrectinib for adults 100 mg bid, for children 100 mg/m^2^ (maximum of 100 mg) bid	ORR (79%)	increased alanine aminotransferase [3%], anemia [2%], decreased neutrophil count [2%]
	Larotrectinib	Drilon A et al., 2018 (38)		CCA with TRK fusion-positive	Larotrectinib for adults 100 mg bid, for children 100 mg/m^2^ (maximum of 100 mg) bid	ORR (75%)	
HER	Erlotinib	Lee J et al., 2012 (39)	1st-line	advanced CCA	Erlotinib 100 mg/d + Gemcitabine 1000 mg/m^2^ + Oxaliplatin 100 mg/m^2^ vs Gemcitabine 1000 mg/m^2^ + Oxaliplatin 100 mg/m^2^	PFS (5.8 m; 4.2 m)	febrile neutropenia [4%]; [6%]
	Cetuximab	Gruenberger B et al., 2010 (40)	1st-line	unresectable locally advanced/metastatic CCA	Cetuximab 500 mg/m^2^ + Gemcitabine 1000 mg/m^2^ + Oxaliplatin 100 mg/m^2^	ORR (63%)	skin rash (n = 4), peripheral neuropathy (n = 4), thrombocytopenia (n = 3), nausea (n = 1), diarrhoea (n = 1), and neutropenia (n = 1)
	Cetuximab	BINGO (41)	1st-line	locally advanced (non-resectable)/metastatic CCA	Gemcitabine (1000 mg/m^2^) + Oxaliplatin (100 mg/m^2^) + Cetuximab (500 mg/m^2^) vs Gemcitabine (1000 mg/m^2^) + Oxaliplatin (100 mg/m^2^)	PFS4 (63%; 54%)	peripheral neuropathy [24%]; [15%], neutropenia [22%]; [16%], increased aminotransferase concentrations [22%]; [15%]
	Lapatinib	Ramanathan RK et al., 2009 (42)		advanced CCA and HCC	Lapatinib 1500 mg qd	ORR (0%; 5%) PFS (1.8 m; 2.3 m)	
	Lapatinib	Peck J et al., 2012 (43)		unresectable advanced CCA	Lapatinib 1500 mg qd	ORR (0%)	
	Pertuzumab and Trastuzumab	MyPathway (44)	2nd-line	metastatic CCA with HER2 amplification/overexpression	Pertuzumab (840 mg loading dose, then 420 mg every 3 weeks) + Trastuzumab (8 mg/kg loading dose, then 6 mg/kg every 3 weeks)	ORR (23%)	alanine aminotransferase increase [13%], aspartate aminotransferase increase [13%]
	Neratinib	SUMMIT (45)		HER2 (ERBB2) mutation-positive advanced CCA	Neratinib 240 mg qd	ORR (12%) CBR (20%) PFS (2.8m)	
	Trastuzumab/Lapatinib/Pertuzumab	Javle M et al., 2015 (46)		advanced GBC/CCA with HER2/neu genetic aberrations or protein overexpression	Trastuzumab/Lapatinib/Pertuzumab	ORR	
VEGF-TKI	Bevacizumab	Zhu AX et al., 2010 (47)		advanced CCA	Bevacizumab 10 mg/kg + Gemcitabine 1000 mg/m^2^ + Oxaliplatin 85 mg/m^2^	PFS (7.0 m)	neutropenia (n = 7), raised alanine aminotransferase concentrations (n = 5), peripheral neuropathy (n = 5), hypertension (n = 5)
	Sorafenib	Bengala C et al., 2010 (48)		advanced CCA	Sorafenib 400 mg bid	12wDCR (32.6%)	
	Sunitinib	Yi JH et al., 2012 (49)	2nd-line	unresectable metastatic CCA	Sunitinib 37.5 mg qd	TTP (1.7m)	Neutropenia [21.4%]、thrombocytopenia [21.4%]
	Ramucirumab、Merestinib	Valle J W et al., 2021 (50)	1st-line	locally advanced or metastatic CCA	Ramucirumab 8 mg/kg + Cisplatin 25 mg/m^2^ + Gemcitabine 1000 mg/m^2^ vs Merestinib 80 mg + Cisplatin 25 mg/m^2^ + Gemcitabine 1000 mg/m^2^ vs placebo + Cisplatin 25 mg/m^2^ + Gemcitabine 1000 mg/m^2^	PFS (6.5m;7.0 m;6.6 m)	Neutropenia ([49%]; [47%]; [33%]), thrombocytopenia ((35%); (19%); [17%])、anaemia ([27%]; (16%); [19%])
PI3K/AKT/mTOR	Copanlisib	Tan E S et al., 2020 (51)	1st-line	advanced/unresectable CCA	Copanlisib + Gemcitabine + Cisplatin	PFS6 (51%)	decreased neutrophil count [45.83%], anemia [25%], increased lipase[25%], hypertension [20.8%]
	Everolimus	EUDRACT 2008-007152-94 (52)	2nd-line	locally advanced/metastatic/recurrent CCA	Everolimus 10 mg qd	DCR (44.7%) ORR (5.1%)	
	Everolimus	RADiChol (53)	1st-line	advanced CCA	Everolimus 10 mg qd	12 w DCR (48%)	

FGFR, fibroblast growth factor receptor; ORR, object response rate; PFS, progression-free survival; cORR, certain objective response rate; mDOR, median duration of response; DCR, disease control rate; IDH1/2, Isocitrate dehydrogenase 1/2; PR, partial response; NTRK, neurotrophin receptor kinase; CBR, clinical benefit rate; TTP, time to progression.

### 2.1 FGFR

The FGFR family of transmembrane receptors has five members (FGFR1–5). FGFR1–4 involve an endocellular tyrosine kinase domain, which triggers signaling *via* multiple pathways when activated, including PI3K/AKT/mTOR, RAS-Raf-MEK-ERK and JAK/STAT signaling pathways which are closely associated with cell proliferation, differentiation, migration, and angiogenesis (54). In addition to the point mutations and gene amplification, the most common genetic alterations of FGFRs in CCA are FGFR gene fusions/rearrangements, mainly in iCCA (55). Farther sequencing researches revealed that FGFR2 fusions were observed in about 14% of iCCA patients, and were generally mutually exclusive with IDH mutations (56).

In a sequencing study involving 115 CCA patients, the mutations in FGFR2 were the most common (6.1%) compared with those in FGFR1 (0.9%) and FGFR3–5 (0%). Although tyrosine kinase inhibitors (TKI) targeting FGFR2 have recently emerged, patients with FGFR2 fusions seem to be the unique group to respond to such inhibitors (12, 23). To date, FGFR2 fusion genes contain FGFR2-AHcyL1, FGFR2-BICC1, FGFR2-MGEA5, FGFR2-PPHLN1, and FRGR2-TACC3 (57). The fusion proteins lead to morphological changes in the cells followed by abnormal cell proliferation. Specific FGFR depressants, such as infigratinib, erdafitinib, pemigatinib, futibatinib, and Debio 1347, reversibly combine with cysteine residues in the P-loop region of the adenosine triphosphate (ATP) pocket (58). Specific FGFR inhibitors have demonstrated clinical efficacy in patients with advanced CCA who are FGFR-fusion-positive. Pemigatinib and infigratinib have been approved by the FDA for use in CCA patients with FGFR2 fusions after prior treatment with standard chemotherapy (59). Pemigatinib, a specific FGFR1/2/3 inhibitor, was the first drug to be authorized for the therapy of terminal CCA (60). In a multicenter phase II clinical study (FighT-202), the ORR was 35.5% within the FGFR2 fusion/rearrangement subgroup and in patients with other FGF/FGFR alterations (12). This study demonstrated the potential therapeutic value of pemigatinib for CCA patients with FGFR2 fusion/rearrangement.

An efficacy study of a second FGFR1/3 inhibitor, infigratinib, was presented at the 2018 ESMO Congress (14). In this study, advanced iCCA patients with FGFR2 gene fusions resistant to standard chemotherapy received oral infigratinib. The preliminary results from that study indicated that the ORR was 31.0% and the certain ORR (cORR) was 26.9%. Moreover, the most common grade 3/4 adverse events (AEs) were hypophosphatemia (14.1%), hyperphosphatemia (12.7%), and hyponatremia (11.3%). This study demonstrated that infigratinib could be used as a therapeutic agent for FGFR2-fused iCCA patients (14). Similarly, a phase II study of gemcitabine-resistant FGFR2 fusion/mutation/amplification in patients with terminal CCA demonstrated controlled toxicity and significant clinical activity with infigratinib (15). In another study, infigratinib was investigated as a first-line treatment in patients with FGFR2-positive advanced CCA in a phase III trial (PROOF 301) (17). Such patients were randomly divided in a 2:1 ratio to accept infigratinib or cisplatin plus gemcitabine. This ongoing study may provide evidence for the first-line FGFR2-targeted treatment in patients with advanced CCA (17).

Another targeted agent investigated for patients with FGFR-positive CCA was derazantinib (61). Apart form prohibiting FGFR, the medicine also blocks other kinases, such as KIT, VEGFR1 and DDR. Mazzaferro et al. evaluated derazantinib in the non-blind phase I/II tests. This study enrolled 29 iCCA patients who did not accept chemotherapy or were not sensitive to chemotherapy. Derazantinib administration resulted in an OS of 20.7% and a DCR of 82.8% (19). Moreover, derazantinib demonstrated significant antitumor activity and controlled toxicity.

A phase IIa study from Asia enrolled 22 CCA patients who received erdafitinib, which is an inhibitor of FGFR1/2/3/4. The ORR, mPFS, and mOS were 40.9%, 5.6 mo, and 40.2 mo, respectively (11). These results indicated that erdafitinib demonstrated significant efficacy and safety in the treatment of advanced CCA with FGFR mutations/fusions. A phase II, open-label, multicenter study indicated that futibatinib, a highly selective and irreversible FGFR1/2/3/4 inhibitor, significantly improved clinical outcomes for the advanced iCCA patients with FGFR2 gene fusion-rearrangement progressing after one or more previous lines of systemic therapy (27).

FGFR2 monotherapy was demonstrated to be more effective and less toxic than conventional chemotherapy and could be regarded as a second-line therapy for terminal CCA. Therefore, it is necessary to routinely evaluate the FGFR fusion/rearrangement in CCA patients. There are several treatments for the side effects of FGFR inhibitors, dietary modification(plant-derived food) and phosphate lowering therapies(phosphate binders and phosphaturic agents) can lower hyperphosphatemia, optimize nutrition and sleep are necessary for relieving fatigue, fluids intake and probiotics supplementcan improve diarrhea symptoms (62).

### 2.2 IDH 1/2 mutations

Among the three subtypes of IDH, IDH1 and IDH2 have significant carcinogenic effects. IDH 1/2 is a protease participated in DNA transcription and reestablish. Generally, this protease facilitates the transformation of isocitrate to α-ketoglutarate. Nevertheless, mutations in this gene may cause epigenetic alterations such as enhanced production of 2-hydroxyglutarate (a tumor metabolite), thereby resulting in DNA disruption and histone methylation.

The mutations in the IDH 1/2 gene exist in around 20–25% of iCCA patients and are virtually absent in patients with other CCA subtypes (63). A 2019 research indicated that IDH1 mutations were present in 13% of the 4214 iCCA patients and 0.8% of the 1123 eCCA patients (64). The IDH-1 mutations are more common than IDH-2 mutations and typically appear in non-hepatitis CCA patients (65).

In a phase III trial (ClarIDHy), ivosidenib, an IDH 1/2 inhibitor, was found to be more effective than a placebo in advanced CCA patients with IDH 1/2 mutations (29). The mPFS improved significantly in the ivosidenib (experimental) group (2.7 mo) compared with the placebo group (1.4 mo). The mOS was 10.3 mo in the ivosidenib group and 7.5 mo in the placebo group (p = 0.09). The most familiar grade 3/4 AE in the ivosidenib group was ascites (7%). Therefore, ivosidenib was authorized by the FDA as a updated treatment for chemotherapy-resistant iCCA patients with IDH 1/2 mutations (66).

### 2.3 RAS-RAF-MEK–ERK pathway

The RAS-RAF-MEK-ERK pathway is the primary signal transduction constituent of the mitogen-activated protein kinase (MAPK) cascade. The activated RAS initiates a downstream phosphorylation cascade, including RAF protein kinase, MEK1/2 kinase, and ERK1/2 kinase, which transmits signals from the intracellular membrane to the nucleus. The nuclear effectors regulate cell proliferation, differentiation, and apoptosis. Thus, the dysregulation of the RAS-Raf-MEK-ERK pathway is often the result of genetic alterations, which can lead to tumorigenesis.

KRAS mutations have been found in all the CCA subtypes but in different proportions and are associated with poorer prognosis (67). Effective RAS inhibitors have not been prescribed for decades (68), and therapies have centered on inhibiting the downstream components of RAS kinase. The most potent activators of this pathway are alterations in the BRAF gene, most commonly caused by a substitution of valine for glutamate (V600E). This mutation occurs at a lower rate (1–6%) in patients with CCA, especially iCCA (55). A recent study involving 54 iCCA patients with a V600E point mutation showed that this mutation was related to advanced TNM stage, chemoresistance, and worse life expectancy (69). However, as BRAF inhibitor monotherapy demonstrated limited efficacy in clinical use, combination therapy should be applied. A phase II trial evaluated the combination of the BRAF inhibitor dabrafenib and the MEK inhibitor trametinib in 178 tumor patients with V600E mutations, involving 33 patients with terminal CCA (31). The ORR was 41%, mPFS was 7.2 mo, and mOS was 11.3 mo. The patients experienced three grade 3/4 AEs, including elevated γ-glutamyltransferase (9%) and decreased white blood cell count (9%). Dabrafenib plus trametinib demonstrated promising efficacy with a favorable safety profile in BRAF V600E mutation-positive CCA patients.

In a phase II trial involving 28 advanced CCA patients, the MEK inhibitor selumetinib demonstrated promising clinical activity, with an ORR of 12%, mPFS of 3.7 mo, and mOS of 9.8 mo. The most common grade 3/4 AE was fatigue (4%) (32). Ulixertinib, the emerging ERK 1/2 depressor, has proved promising clinical effectivity in patients with MAPK-driven terminal tumors (35).

### 2.4 NTRK

NTRKs are a group of tyrosine kinases comprising of three portions (NTRK-1/2/3) that contribute to tumorigenesis in a large number of cancer patients through oncogenic fusion resulting in structural activation (70). Such fusions have been reported in CCA (36), with a chimeric NTRK gene spotted in 3.5% of the iCCA patients. The NTRK gene encodes the tropomyosin receptor kinase (TRK) receptor, which is activated by the chimeric gene for the promotion of differentiation, proliferation, and survival of cancer cells. Larotrectinib, a TRK receptor inhibitor, was currently approved by the FDA and EMA (37). In a phase II trial,55 patients with TRK fusion-positive advanced eCCA were administered larotrectinib. The ORR was found to be 75% (38).

### 2.5 HER

The four members of the epidermal growth factor receptor family (HER1/2/3/4) are tyrosine kinases receptors that are activated by diverse ligands to become homodimers or heterodimers. EGFR overexpression significantly enhanced the incidence of microvascular infiltration, lymph node metastasis, and perineural invasion (71). EGFR and HER2 overexpression are considered to be independent poor prognostic factors in CCA (72). The downstream pathways activated by the EGFR family members include RAS-RAF-MEK-ERK, PI3K/AKT/mTOR, and JAK/STAT (73). The expression of the EGFR pathway is prevalent in CCA, and a previous study demonstrated that it was expressed in 100% iCCA, 52.6% eCCA, and 38.5% GBC cases (74).

California Consortium conducted a phase II study of lapatinib, a dual HER2 and EGFR inhibitor, indicating lapatinib is well tolerated and effective in HCC patients but demonstrated minimal effect in CCA patients (42). Another phase II study evaluated the efficacy and safety of lapatinib in 25 advanced CCA patients (43). No somatic mutations in EGFR or HER2/neu were observed in enrolled patients. Lapatinib was well tolerated but failed to demonstrate significant efficacy as a monotherapy for CCA, indicating that targeting HER2 did not seem to be an effective approach for CCA. However, another study involving 14 GBC patients with HER2 overexpression or mutation as well as one eCCA patient demonstrated significant clinical activity with the targeted use of HER2 inhibitors (75).

Heterodimers containing HER2 are highly potent signal transducers. HER2 (ERBB2) mediates its signal transduction *via* MAPK and PI3K pathways. HER2 mutations are observed in approximately 5–15% of CCA cases, most commonly in GBC and eCCA (76). The potential molecular targets include HER2 alterations, HER2 amplification, and HER2 overexpression. The targeted therapy with anti-HER2 agents such as pertuzumab and trastuzumab has significantly improved the prognosis of CCA patients (44, 46).

MyPathway, multicenter phase IIa, prospective trial, evaluated the efficacy of pertuzumab + trastuzumab-targeted therapy in 39 advanced CCA patients with HER2 amplification and/or overexpression (44). The results demonstrated that the combination of pertuzumab and trastuzumab was effective and well tolerated. Another phase II SUMMIT trial evaluated the efficacy and safety of neratinib, an irreversible HER1/2/4 inhibitor, in 25 advanced CCA with HER2 mutations. The ORR was 12%, mPFS was 2.8 mo, and mOS was 5.4 mo. The most common treatment-related AEs were diarrhea (56%) and vomiting (48%) (45). In a retrospective study (46), the efficacy and safety of trastuzumab, lapatinib, and pertuzumab were analyzed in patients with HER2-positive advanced GBC/CCA. HER2 amplification or overexpression was observed in 8 patients in the GBC group, with stable disease (n = 3), partial response (PR) (n = 4), or complete response (n = 1). These results indicated that HER2 inhibition is a promising therapeutic strategy for GBC patients with HER2 gene amplification and deserves further exploration.

### 2.6 VEGF-TKI

Angiogenesis plays an important part in maintaining tumor survival, and the microvessel density (MVD) measurements indicate high vascularization in eCCA. High MVD is associated with higher lymph node diffusivity and postoperative local recurrence rates, thereby indicating poor prognosis of eCCA and other CCA subtypes. The vascular endothelial growth factor (VEGF) family and its receptors not only mediate tumor invasion by affecting angiogenesis and vascular permeability but also mediate tumorigenesis through the cell signal transduction pathway. The VEGF expression is correlated with tumor grade, invasion ability, and prognosis.

The VEGF inhibitors include VEGF monoclonal antibodies (mAbs) and multireceptor TKIs. Bevacizumab binds and neutralizes VEGF-A ligands and has little benefit as a monotherapy because of commutative activation mechanisms, that attenuate the antiangiogenic activity of VEGF inhibition. Nevertheless, TKIs can not only block multiple signaling pathways, including angiogenesis but are also directly involved in tumor growth.

Sorafenib, a multikinase inhibitor of VEGFR-2/3, PDGFR-β, b-RAF, and c-Raf, has demonstrated activity in preclinical models of CCA. In a phase II trial conducted by Bengala et al. (48) involving 46 advanced CCA patients, administration of single-agent sorafenib demonstrated less efficacy with manageable adverse reactions. Another phase II study evaluated the safety and efficiency of sunitinib as a second-line therapy for 56 advanced CCA patients with prior treatment with chemotherapy. The results from that study indicated that the median time to progression (mTTP) was 1.7 mo and the ORR and DCR were 8.9% and 50.0%, respectively. The most common grade 3/4 toxic side effects were neutropenia and thrombocytopenia (21.4%) (49).

### 2.7 PI3K/AKT/mTOR

The PI3K/AKT/mTOR signaling pathway plays an important part in cell proliferation and survival and participated in the development and progression of CCA. In a study (77) involving 88 CCAs (66% eCCA), activated mTOR was found to be a negative prognostic indicator (78). In addition, the upregulation of mTOR downstream effectors is common in CCA.

In a phase II trial (53) involving 39 advanced CCA patients who had received prior chemotherapy, the mTOR inhibitor everolimus demonstrated promising anti-tumor efficacy with tolerable toxic and side effects. Furthermore, the RADiChol study involving 27 advanced CCA patients indicated that everolimus as first-line monotherapy demonstrated promising clinical efficacy.

### 2.8 PARP

Poly ADP-ribose polymerase (PARP) depressants are used in the treatment of tumors with homologous repair defects (HRD) for targeting tumor cells with DNA damage repair (DDR) defects. The lack of a functional PARP enzyme results in DNA double-stranded breaks and consequent cell death. The alterations in several DDR genes may contribute to HRD, especially IDH1 and IDH2 mutations, most commonly observed in eCCA (79). Multiple phase II trials are underway for evaluating the PARP depressants in CCA, including monotherapy(NCT04042831, NCT03207347, NCT03212274) and combination with PD-1/PD-L1 inhibitors(NCT04895046, NCT04306367) or temozolomide(NCT04796454), demonstrating considerable efficacy.

Besides, Dr. Furuse et al. discussed another targeted drug, Nanvuranlat, an L-type amino acid transporter (LAT1) inhibitor for patients with pretreated advanced refractory CCA in ASCO GI 2023, showing that it has certain therapeutic effect.

## 3 Immunotherapy

Based on the immune microenvironment (TME) of CCA, the latest approaches to immunotherapy for CCA patients, including preclinical and clinical studies of immune checkpoint inhibitors (ICIs), cancer vaccines, and adoptive cell therapy are summarized in **Table 2**.

**Table 2:** Selected clinical trials of immunotherapy in advanced cholangiocarcinoma.

Mutation/Pathway	Agent	Trial	Setting	Patients	TreatmentArm(s)	Primary Endpoint	Grade 3/4 Adverse Events
PD-1/PD-L1	Nivolumab	Ueno M et al., 2019 (80)		unresectable/recurrent CCA	Nivolumab 240 mg q2w vs Nivolumab 240 mg q2w + Cisplatin 25 mg/m^2^ + Gemcitabine 1000 mg/m^2^	OS (5.2 m;15.4 m) PFS (1.4 m; 4.2 m)	neutrophil count decrease [77%], platelet count decrease [50%]
	Nivolumab	Kim RD et al., 2020 (7)	2nd-line	advanced refractory CCA	Nivolumab 240 mg q2w for 16 weeks, then 480 mg q4w	ORR (22%)	
	Pembrolizumab	KEYNOTE-158 (81)	2nd-line	advanced/metastatic CCA	Pembrolizumab 200 mg Q3W	ORR (5.8%)	renal failure (n = 1), immune-mediated AE, or infusion reaction [18.3%]
	Pembrolizumab	KEYNOTE-028 (81)	2nd-line	advanced/metastatic CCA with PD-L1-positive	Pembrolizumab 10 mg/kg Q2W	ORR (13.0%)	immune-mediated AE or infusion reaction [20.8%]
	Pembrolizumab	Lee S H et al., 2020 (82)	2nd-line	CisGem-refractory CCA with PD-L1–positive	Pembrolizumab 200 mg q3w	ORR (9.8%); mPFS (2.1 m); OS (6.9 m)	
	Pembrolizumab	Kang J et al., 2020 (83)	2nd-line	CisGem-refractory advanced CCA with PD-L1–positive	Pembrolizumab 200 mg q3w	ORR (10%); mPFS (1.5 m); OS (4.3 m)	
	Durvalumab 、Tremelimumab	Ioka T et al., 2019 (84)	2nd-line	advanced CCA	Durvalumab 10 mg/kg q2w vs Durvalumab 20 mg/kg + Tremelimumab 1.0 mg/kg q4w	mOS (8.1 m;10.1 m)	
	Durvalumab	TOPAZ-1 (85)	1st-line	advanced CCA	Durvalumab (1500 mg Q3W) or placebo + CisGem (Gem 1000 mg/m^2^ and Cis 25 mg/m^2^ on days 1 and 8 Q3W), followed by Durvalumab (1500 mg Q4W) or placebo	OS (p = 0.021)	
	Atezolizumab	Yarchoan M et al., 2020 (10)	2nd-line	advanced CCA	Atezolizumab 840mg q2w vs Cobimetinib 60 mg qd (21 days on/7 days off) + Atezolizumab	PFS (1.87 m; 3.65 m)	
	Atezolizumab	IMbrave 151 (86)	1st-line	Advanced CCA	Atezolizumab 1200mg q3w + bevacizumab 15mg/kg q3w + CisGem (Gem 1000 mg/m2 and Cis 25 mg/m2 on days 1 and 8 q3W) vs atezolizumab + placebo + Gem/Cis	PFS(8.3m; 7.9m)	
	Pembrolizumab	KEYNOTE-158 (87)	2nd-line	advanced MSI-H/dMMR non colorectal cancer	Pembrolizumab 200 mg q3w	ORR (34.3%)	pneumonia [0.4%]
	Pembrolizumab	Marcus, L et al., 2019 (88)	2nd-line	unresectable/metastatic MSI-H/dMMR solid tumors	Pembrolizumab 10 mg/kg q2w/200 mg q3w	ORR (39.6%)	
CAR T cell	CART-EGFR cell	Guo Y et al., 2018 (89)	2nd-line	EGFR-positive advanced unresectable/relapsed/metastatic CCA	median CART cell dose: 2.65 × 106/kg	mPFS (4 m)	acute fever/chill, lymphopenia, and thrombocytopenia

OS, overall survival; CART, chimeric antigen receptor T cell.

### 3.1 Immune checkpoint inhibitors

Studies have indicated PD-L1 expression in about half of the CCA patients, which is associated with poor prognosis (7). As studies have revealed that ICCs such as PD-1/PDL1 and CTLA-4 adjust antitumoral immune reactions, particular depressants against these targets have been exploited and used in anticancer therapy.

A multicenter phase II study involving 54 patients evaluated the efficacy and safety of nivolumab monotherapy in advanced CCA patients after the first- to third-line of treatment (7). The results revealed that the ORR was 22% and DCR 59%. In addition, the mPFS and mOS were 3.68 mo and 14.24 mo, respectively. They further indicated that the PD-L1 expression in tumors was positive in all the patients who responded to treatment and was associated with prolonged PFS. Similarly, a multicenter retrospective study was conducted for evaluating the clinical efficacy and safety of pembrolizumab in CisGem-refractory CCA patients (82). 51 advanced CCA patients who were PD-L1-positive after progression on first-line CisGem progression were enrolled and administered pembrolizumab. The ORR was 9.8%, mPFS was 2.1 mo, and OS was 6.9 mo. The grade 3/4 AEs occurred in only 4 patients (7.8%).

A phase I study evaluated the safety and tolerability of durvalumab (anti-PD-L1 antibody) and tremelimumab (anti-CTLA-4 antibody) in advanced CCA patients who had progressed on previous therapy (84). PR was existed in 2 patients in the durvalumab group and 7 patients in the durvalumab plus tremelimumab group. The mPFS and mOS were 8.1 mo and 10.1 mo, respectively. That study demonstrated that both durvalumab monotherapy and durvalumab plus tremelimumab combination therapy can be tolerated, and demonstrated good clinical efficacy.

Both germline mutations due to DNA mismatch repair (MMR) and somatic hypermethylation of the MLH1 promoter can lead to MMR defects (dMMR), which, in turn, can lead to MSI-H status in Lynch syndrome patients (89). The somatic mutation rates of MSI-H cancers are one to two orders of magnitude higher than those of microsatellite-stable cancers, with enhanced neoepitope production, intense lymphocytic infiltration, and a better prognosis, indicating that the PD-1/PD-L1 inhibitors can be used in the treatment of MSI-H cancers (90). MSI-H rarely occurs in patients with CCA and is more common in patients with eCCA. In a Japanese study (91), 13.2% of 38 eCCA patients had MSI-H. There have been several reports of partial responses in MSI-H CCA patients received with the PD-1 inhibitor pembrolizumab, including one study in eCCA patients that lasted more than 13 months (92).

In the KEYNOTE-158 trial involving 233 patients with 27 tumor types (endometrial, gastric, cholangiocarcinoma, and pancreatic cancer), including 22 patients with advanced CCA, pembrolizumab was used in pretreated MSI-H/dMMR cancer patients. The ORR was 34.3%, mPFS was 4.1 mo, and mOS was 23.5 mo. The grade 3–5 treatment-related AEs occurred in 34 patients (14.6%) (93). The studies have indicated that pembrolizumab had significant clinical benefits and manageable toxicity in previously treated advanced MSI-H/dMMR non-colorectal cancer patients. The FDA approved pembrolizumab as a second-line therapy for patients with advanced MSI-H/dMMR cancer (88).

### 3.2 Tumor-infiltrating leukocyte

The future role of immunotherapy in ICC is still unknown. A study involving 68 resectable ICC patients who underwent complete resection was conducted for evaluating the predictive value of TILs on the OS and PFS in patients. TIL was an independent negative predictor of poor OS and poor PFS. Low TIL was associated with high levels of CA125 and CA19-9 (94).

In a study conducted to analyze the tissue characteristics of tumor-invasive CD8+ T cells in ICC patients (95), blood and tissue samples were collected from 33 HCC patients. The results indicated that CD69+ CD103+ TRM-like CD8+ TILs represent significant tumor-specific immune responses. It is expected to be a potential therapeutic target in ICC patients.

### 3.3 Cancer vaccines

If the tumor cells are considered a “pathogen” with tumor-specific antigens, a vaccine injected with these antigens can stimulate the immune system and facilitate the differentiation of memory T cells, thereby increasing the anti-tumor immune anility. Several types of cancer vaccines, including single peptidyl, polypeptidyl, and dendritic cell (DC) vaccines, have been developed. However, a retrospective study involving advanced CCA patients demonstrated a PR of 6%, stable disease (SD) of 23%, and ORR of only 6% (8). It is speculated that weak anti-tumor responses of tumor vaccines are observed in CCA because of TME-regulated immunosuppression. Therefore, merely a fraction of T cells sensitive to the vaccine. Moreover, activatory T cells have trouble in tumor penetration as a result of the T-cell rejection mechanism described above. Therefore, the association of ICIs and tumor vaccines may represent the future of cancer vaccines.

### 3.4 Adoptive immunotherapy

Since the TME of CCA can inhibit T cell activity and evade the immune system, artificial injection of invasive cancer-specific T cells into the tumors may provide a novel approach for antitumor therapy. Fourth-generation anti-CD133CAR T cells demonstrated promising effectiveness against CD133-expressing CCA cells (96). Feng et al. showed an advanced CCA patient who was treated with EGFR and CD133-directed CAR-T cocktail of immunotherapy (9). A PR of 8.5 mo was achieved with EGFR-targeted CAR-T cells and 4.5 months with CART-133. A phase I trial of EGFR-specific CAR-T cells for the treatment of EGFR-positive terminal CCA demonstrated promising outcomes as well, with 58.8% achieving SD and 5.8% of patients achieving a CR (89). However, considering the toxicity and endothelial damage caused by CAR-T cells, further researches are necessary to demonstrate the safety and effectiveness of this treatment.

### 3.5 Oncolytic viruses

The oncolytic virus (OV) therapy infects and kills cancer cells through the natural or acquired ability of the viruses. In the past few years, in-depth research on viral gene function and molecular biology has meaningfully advanced the effectiveness and safety of OVs. The recruitment of T cells and the induction of tumor-reactive immunity form the basis of OV therapy. The researches have showed that OVs can enhance the immune system response after infecting the tumor cells. Currently, Talimogene laherparepvec is the merely OV authorized in the United States (97). Furthermore, the application of OV therapy in association with the inhibitors of the transforming growth factor-beta signal pathway could facilitate the effectiveness of immunotherapy. The objectives of the OV therapy include enhancing the alternative duplication power of OVs, selecting an efficient mode of OV infection, finding a equilibrium between the antiviral and the antitumor immunity, removing immunosuppressive TME, and increaseing the oncolytic effect. The oncolytic roles of a measles vaccine virus and three survitin-based conditionally replicating adenoviruses for CCA treatment were demonstrated in a preclinical study (98).

## 4 Combination therapy

### 4.1 Combination of targeted therapy and chemotherapy

In the past few years, several studies aim to evaluate the efficacy of the combination of targeted therapy and chemotherapy. However, these studies have shown contrasting results. ABC-04 study (33) enrolled 8 advanced CCA patients who received selumetinib in combination with CisGem as the first-line therapy. Among these patients, 3 had a PR and 5 had stable disease, with an mPFS of 6.4 mo. Subsequently, the BIL-MEK study involved 57 advanced CCA patients who were previously untreated. This study evaluated the efficacy of a sequential combination of selumetinib followed by CisGem. However, irrespective of the dosing regimen, this combination did not improve the tumor response at 10 weeks or prolong survival but instead increased toxicity (34).

A randomized phase III study comparing gemcitabine plus oxaliplatin (GEMOX) plus erlotinib versus GEMOX as the first-line therapy in advanced CCA patients (39). Distinct differences were not observed in the PFS and mOS between the two groups (p = 0.087; 0.611 respectively). The ORR of the GEMOX plus erlotinib group was higher than that of the GEMOX group (29.6% vs 15.8%; p = 0.005). The most common grade 3/4 AE was febrile neutropenia. GEMOX plus erlotinib demonstrated significant antitumor activity and could be used as a therapy modality in terminal CCA patients.

In a phase II research (40) involving 30 terminal CCA patients, cetuximab plus GEMOX was used as the first-line therapy. The results revealed that mPFS was 8.3 mo, OS was 12.7 mo, and ORR was 63% (complete response (CR) was 10%). Moreover, no grade 4 AEs occurred in that study. These results indicated that cetuximab plus GEMOX was well tolerated and demonstrated significant antitumor activity. Another randomized clinical trial (RCT) investigated the efficacy of cetuximab combined with chemotherapy as first-line therapy in advanced CCA patients, and the results demonstrated that the combination of GEMOX and cetuximab did not enhance the efficacy of chemotherapy when compared with GEMOX monotherapy in advanced CCA patients, although it was well tolerated (41).

In a recent phase II trial (51), the PI3K suppressant copanlisib was applied as second-line therapy in association with CisGem in advanced CCA patients. The results implied that mPFS and mOS were 6.2 mo and 13.7 mo, respectively. Some studies investigated the efficacy of the VEGF inhibitors in combination with GEMOX therapy for advanced CCA patients and showed little evidence of any benefit (47) (50). Presently, a phase III study of Fight-302 comparing pemigatinib with gemcitabine plus cisplatin (CisGem) as a first-line treatment for advanced CCA with FGFR2 gene rearrangement is in progress(NCT03656536). The primary endpoint of this study is PFS, and the secondary endpoints are ORR, OS, and DCR (13).

### 4.2 Combination of immunotherapy and chemotherapy

In a multicenter phase I study conducted in Japan, the safety and tolerability of the PD-1 ICI nivolumab alone or in combination with chemotherapy were evaluated in advanced CCA (80). Thirty patients were enrolled in each group, with advanced CCA patients who had progressed on previous gemcitabine therapy receiving nivolumab monotherapy and advanced CCA patients without prior treatment with chemotherapy receiving nivolumab plus Cis plus Gem. In the nivolumab group, mOS was 5.2 mo and mPFS was 1.4 mo. In the nivolumab plus CisGem group, mOS was 15.4 mo and mPFS was 4.2 mo. The most common side effects in the nivolumab group were decreased appetite (17%), fatigue (13%), and pruritus (13%). In the nivolumab plus CisGem group, the most common side effects were a decrease in neutrophil count (83%) and platelet count (83%). The results indicated that nivolumab demonstrated significant clinical efficacy and controllable toxic and side effects.

Topaz-1 was the first global phase III study evaluating first-line durvalumab plus CisGem in advanced CCA treatment (85). In this double-blind study, 685 advanced CCA patients who had no prior treatment were randomly assigned 1:1 to receive durvalumab plus CisGem (n = 341) or placebo plus CisGem (n = 344). The OS and PFS were significantly improved with durvalumab plus CisGem compared with placebo plus CisGem. The grade 3/4 AEs occurred in 62.7% of patients of the durvalumab group and 64.9% of patients of the placebo group, indicating that durvalumab plus CisGem may become a new first-line standard of care. At present, a phase III randomized KEYNOTE-966 study is in progress for evaluating the effect of pembrolizumab under the same conditions (NCT04003636).

A single-center phase II study involving 128 advanced ICC patients who had not received chemotherapy evaluated gemcitabine and cisplatin plus durvalumab with or without tremelimumab as the first-line treatment in patients with advanced biliary tract cancer. The results from that study indicated that the mOS was 18.1 mo in the gemcitabine plus durvalumab group and 20.7 mo in the gemcitabine plus durvalumab plus tremelimumab group. The combination of gemcitabine, durvalumab, and tremelimumab seems to be a better first-line treatment (99).

### 4.3 Combination of immunotherapy and targeted therapy

The MEK inhibitor monotherapy has limited efficacy in CCA, but it is considered to enhance the effect of PD-L1 inhibitors. Recently, a randomized, multicenter phase II study indicated that a association of atezolizumab (anti-PD-L1 inhibitor) and cobimetinib (MEK inhibitor) was effective in advanced CCA patients with prior first-line or second-line treatment. The mPFS was 3.65 mo in the combination group and 1.87 mo in the single drug group. PR was achieved in only one patient (3.3%) in the combination group and one patient (2.8%) in the single drug group. These results indicated that the combination of drugs could lead to greater clinical benefits. However, the combination group was characterized by more rashes, gastrointestinal events, elevated CPK, and thrombocytopenia (10). Therefore, additional phase III studies comparing ICIs with standard chemotherapy are needed.

### 4.4 Combination of immunotherapy, targeted therapy and chemotherapy

A phase 2 clinical study presented at the Annual Meeting of ESMO in 2020, evaluated the efficacy of a combination of PD-1 inhibitor (toripalimab), target drug (lenvatinib), and GEMOX as the first-line treatment of advanced and unresectable ICC, and obtained excellent results. The ORR was up to 80% and DCR was up to 93.3%, and the 6-month survival rate was as high as 90%. Most of the patients in that study had tumor shrinkage, indicating that more inoperable patients can become operable, which provides a novel idea for the conversion therapy of advanced ICC.

Based on data presented during the Gastrointestinal Cancers Symposium 2023, the Phase 2 IMbrave 151 trial (NCT04677504) found that in patients with advanced CCA, treatment with or without bevacizumab in combination with atrilizumab and CisGem had modest clinical benefits (86).

## 5 Conclusions and prospect

Research on the therapies for CCA is rapidly evolving. However, some important questions regarding the treatment for CCA still remain to be addressed. First, the efficacy of targeted therapies is largely limited by acquired resistance, such as the occurrence of secondary polyclonal mutations (20, 21). However, liquid biopsies can be applied for tracking the emergence of polyclonal mutations and guiding medication (100). Second, the use of a safe and effective combination of chemotherapy, targeted therapy, and immunotherapy still needs to be evaluated (12, 23). Third, the lack of specific predictive and reactive biomarkers has led to studies on genetic screening and microenvironmental testing. The comprehensive mechanisms governing CCA are complex. In addition to the above-mentioned drug action pathways, DNA damage repair (DDR), p53, and MDM2 oncoprotein have also been evaluated as therapeutic options in recent studies. Therefore, further larger clinical studies are necessary to validate the safety and effectiveness of different CCA treatment options. Targeted therapy and immunotherapy have demonstrated promising results, and these drugs are expected to be used as first-line therapies in the future.

## Author contributions

EZ contributed to the conception of the study, JD contributed significantly to analysis and manuscript preparation, XL and ZZ performed the data analyses and wrote the manuscript, ZH and EZ helped perform the analysis with constructive discussions. All authors contributed to the article and approved the submitted version.
